# Chaotic dynamics and control in a resonance-mediated acoustic droplet vaporization model

**DOI:** 10.21203/rs.3.rs-11066526/v1

**Published:** 2026-10-15

**Authors:** Tianye Zhang, Ge Wang, Chenzhe Wang, Jianyu Hua, Chengzhi Shi

## Abstract

Acoustic droplet vaporization (ADV) is increasingly used in biomedical research, yet whether its underlying liquid-droplet dynamics under high driving pressure can become chaotic remains unresolved. Such complex dynamics could impair predictable activation and control of imaging and therapeutic responses. Here, we report, to our knowledge, the first evidence of deterministic chaotic dynamics in a liquid-droplet model of resonance-mediated ADV and demonstrate their control by using a weak second harmonic force. An irregular phase portrait, a broadband spectrum, and a positive Lyapunov exponent indicate chaos, and the bifurcation diagram traces the route from period-doubling to chaos. Furthermore, a second harmonic force with 4% of the primary pressure amplitude is applied to reconstruct a period-eight orbit by controlling chaos. Switching the second harmonic force on and off resolves the approach to recurrence and the control of chaotic motion. These results extend ADV resonance theory from amplification to chaotic dynamics and identify chaos control with little loss of radial excursion. The liquid-state droplet model predicts that chaos control can improve the harmonic ADV activation for nonlinear acoustic applications.

